# Cerebral hemodynamics and optic nerve sheath diameter acquired via neurosonology in critical patients with severe coronavirus disease: experience of a national referral hospital in Peru

**DOI:** 10.3389/fneur.2024.1340749

**Published:** 2024-05-03

**Authors:** Omar Heredia-Orbegoso, Miguel A. Vences, Virgilio E. Failoc-Rojas, Diana Fernández-Merjildo, Richard H. Lainez-Chacon, Renán Villamonte

**Affiliations:** ^1^Centro de Emergencia de Lima Metropolitana, Hospital Nacional Edgardo Rebagliati Martins, Unidad de Cuidados Intensivos, Lima, Peru; ^2^Escuela de Medicina, Universidad César Vallejo, Piura, Peru; ^3^Universidad San Ignacio de Loyola, Lima, Peru; ^4^Hospital Cayetano Heredia, Lima, Peru

**Keywords:** COVID-19, ultrasonography, Doppler, transcranial, optic nerve, intracranial hypertension, mortality

## Abstract

**Aim:**

We aimed to describe the neurosonological findings related to cerebral hemodynamics acquired using transcranial Doppler and to determine the frequency of elevated ICP by optic nerve sheath diameter (ONSD) measurement in patients with severe coronavirus disease (COVID-19) hospitalized in the intensive care unit of a national referral hospital in Peru.

**Methods:**

We included a retrospective cohort of adult patients hospitalized with severe COVID-19 and acute respiratory failure within the first 7 days of mechanical ventilation under deep sedoanalgesia, with or without neuromuscular blockade who underwent ocular ultrasound and transcranial Doppler. We determine the frequency of elevated ICP by measuring the diameter of the optic nerve sheath, choosing as best cut-off value a diameter equal to or >5.8 mm. We also determine the frequency of sonographic patterns obtained by transcranial Doppler. Through insonation of the middle cerebral artery. Likewise, we evaluated the associations of clinical, mechanical ventilator, and arterial blood gas variables with ONSD ≥5.8 mm and pulsatility index (PI) ≥1.1. We also evaluated the associations of hemodynamic findings and ONSD with mortality the effect size was estimated using Poisson regression models with robust variance.

**Results:**

This study included 142 patients. The mean age was 51.39 ± 13.3 years, and 78.9% of patients were male. Vasopressors were used in 45.1% of patients, and mean arterial pressure was 81.87 ± 10.64 mmHg. The mean partial pressure of carbon dioxide (PaCO_2_) was elevated (54.08 ± 16.01 mmHg). Elevated intracranial pressure was seen in 83.1% of patients, as estimated based on ONSD ≥5.8 mm. A mortality rate of 16.2% was reported. In the multivariate analysis, age was associated with elevated ONSD (risk ratio [RR] = 1.07). PaCO_2_ was a protective factor (RR = 0.64) in the cases of PI ≥ 1.1. In the mortality analysis, the mean velocity was a risk factor for mortality (RR = 1.15).

**Conclusions:**

A high rate of intracranial hypertension was reported, with ONSD measurement being the most reliable method for estimation. The increase in ICP measured by ONSD in patients with severe COVID-19 on mechanical ventilation is not associated to hypercapnia or elevated intrathoracic pressures derived from protective mechanical ventilation.

## Introduction

The coronavirus disease (COVID-19) pandemic was declared on March 11, 2020 (1), and it was not until May 5, 2023, that the World Health Organization (WHO) announced that the global health emergency caused by this disease had ended (2). In recent years, it has been shown that severe acute respiratory syndrome coronavirus 2 (SARS-CoV-2) infection not only involves the respiratory system but also other organs and systems, such as the nervous system (3).

Early studies on the neurological manifestations of COVID-19 reported on the involvement of the central and peripheral nervous system in up to 36% of patients. The reported symptoms included dizziness, headache, delirium, stroke, altered taste, and altered smell, among other manifestations (3).

Neurological manifestations have also been reported in patients with acute respiratory failure on mechanical ventilation owing to severe COVID-19, and delirium was the main neurological manifestation that was described (4). Critically ill patients with severe SARS-CoV-2 infection require elevated oxygen and ventilatory and hemodynamic support; therefore, deep sedation and neuromuscular blockade are necessary (5–7).

This critical condition limits the neurological evaluation of these patients to pupillary examination and imaging modalities, such as computed tomography (CT) or magnetic resonance imaging (MRI); these examinations require transfer to imaging rooms and the results are complicated hindered by patients being placed in respiratory isolation and their hemodynamic status (8–10).

Neurosonology techniques have proven to be useful, practical, and noninvasive techniques that can be used in critically ill patients. The main advantage of these techniques is the possibility of performing them without moving the patient (11–14).

Transcranial Doppler (TCD) is a non-invasive technique that provides real-time measurement of blood flow velocity in the cerebral arteries, which correlates with cerebral blood flow (CBF), allowing for the assessment of cerebral hemodynamics in critically ill patients. With TCD, the use of invasive CBF measurement techniques can be avoided, while similar prognostic information can be acquired (15, 16).

Optic nerve sheath diameter (ONSD) measurement is an established technique for the assessment of intracranial pressure (ICP) and detection of intracranial hypertension in critically ill patients. It is widely available, cost-effective, time-effective, and non-invasive and does not require additional resources. Therefore, an extended ONSD is a robust predictor of elevated ICP (17–19).

The aim of the present study was to determine the frequency of elevated ICP by optic nerve sheath diameter (ONSD) measurement and describe the neurosonological findings related to cerebral hemodynamics acquired via TCD in patients with severe COVID-19 hospitalized in the intensive care unit of a national referral hospital in Peru.

## Methods

### Design and population

This retrospective cohort study was conducted in the critical respiratory unit of a national referral hospital in Peru, which is intended exclusively for the care of critically ill patients diagnosed with COVID-19.

The study population included patients older than 18 years, who were hospitalized in the intensive care unit of the emergency department during the COVID-19 pandemic in Peru from May 23 to December 31, 2020; these patients were diagnosed with severe COVID-19 and acute respiratory failure, and they were included within the first 7 days of mechanical ventilation under deep sedoanalgesia, with or without neuromuscular blockade.

Patients with a clinical manifestation compatible with that of COVID-19 and positive rapid serological test (immunoglobulin M or G) or nasopharyngeal swab reverse transcriptase polymerase chain reaction (RT-PCR) test were considered as COVID-19 patients.

The severity criteria of the Infectious Diseases Society of America/American Thoracic Society were used to determine the severity of COVID-19 pneumonia (20): Patients with COVID-19 and Acute Respiratory Failure on Mechanical Ventilation with Hypoxemia PaO2/FiO2 ≤ 300 + PEEP ≥ 5 cmH2O and Chest CT bilateral with peripheral, and sub-pleural or peribroncho-vascular areas of patchy or segmental pure ground-glass opacities with the involvement of lower lobes, rare consolidations, reverse halo sign and vascular dilatation.

Patients with hemodynamic instability, those with hypotension (mean arterial pressure [MAP] < 60 mmHg) despite the use of vasopressors, and those with refractory hypoxemia (arterial oxygen partial pressure [PaO_2_] < 40 mmHg with fractional inspired oxygen [FiO_2_] = 100%) were excluded.

Non-probabilistic consecutive selections were performed, which included the entire universe of patients in the study during the established period, resulting in a total of 142 selected participants. Information was collected from the enrolled patients' clinical history (physical or electronic) recorded from admission to discharge or death.

The study was approved by the ethics committee of our institution, formed exclusively to evaluate research on COVID 19.

### Variables

Demographic data (age and sex) and vital function at admission were recorded. The systolic blood pressure (SBP), diastolic blood pressure (DBP) and mean arterial pressure (MAP) values were obtained and recorded from a multiparameter monitor using an invasive arterial catheter. Similarly, the end tidal carbon dioxide (etCO_2_) levels and temperature values were recorded at the same time as sonographic studies. During the hospital follow-up, mortality outcomes (discharge or death) were recorded.

Mechanical ventilator parameters such as positive end-expiratory pressure (PEEP), peak airway pressure (P_peak_), plateau pressure (P_plateau_), mean airway pressure (P_mean_), and pulmonary compliance (Cst) were measured and recorded just before starting the sonographic studies.

Blood samples were obtained from arterial lines for arterial gas analysis. The hemoglobin concentration was obtained and recorded from first blood test performed in ICU. Arterial blood gases were obtained simultaneously with ocular ultrasound and transcranial Doppler. Arterial oxygen partial pressure (PaO_2_), arterial carbon dioxide partial pressure (PaCO_2_), arterial oxygen saturation (SatO_2_), and pH were measured and recorded, these samples were analyzed immediately.

To obtain information about neurological compromise, neurosonology was performed as a routine practice in all intubated patients with severe COVID-19 hospitalized in the intensive care unit. Sonological measurement in our patient cohort was done when patients consistently achieved SpO_2_ > 92% and/or MAP > 60 mmHg on the multiparametric monitor, regardless of FiO_2_ levels and required vasopressor doses, all of this to avoid the influence of hypoxemia and hypotension in the results obtained from neurosonology studies. No daily evaluations of ONSD and TCD were done, only a single measurement was performed, first the transcranial Doppler was performed and immediately after the ONSD. Normally this measurement was made between 24 to 48 h after intubation and mechanical ventilation. No patient received anti-cerebral edema measures before the neurosonology studies. At the time of performing the ultrasound evaluations, all our patients were in a supine position to avoid the influence of the prone position on ONSD and TCD.

Sonographic examinations were conducted using an ATYS MEDICAL LOOKI Transcranial Doppler equipped with a 2 MHz transducer and a General Electric Vivid T8 Ultrasound Machine equipped with a 6.0–13.0 MHz linear probe used for ocular ultrasound. The Sonographic examinations were done by one experienced sonographer trained in ocular sonography and transcranial Doppler.

The Transcranial Doppler (TCD) exams were performed in the middle cerebral arteries and the peak systolic velocity (PSV), end-diastolic velocity (EDV), mean velocity (MFV), and pulsatility index (PI) were measured and recorded. The middle cerebral artery was identified through the temporal window with an insonation depth at 40–65 mm from the skull surface. Immediately after TCD measurements, optic nerve studies were performed. The ONSD was measured 3 mm posterior to the papilla. The ONSD recorded was the mean value obtained from at least 3 repeated measurements in both eyes.

### Statistical analysis

The information was recorded in a Microsoft Excel (2016) database and was exported to STATA v17. In brief, categorical variables were expressed as frequency and percentage, while normally distributed quantitative variables were described as mean and standard deviation; partial pressure of carbon dioxide (PaCO_2_), PaO_2_, ONSD, and lung compliance were not normally distributed and were, therefore, presented as median and interquartile range.

As the proportion of missing data was null for most variables, no statistical artifacts were used to deal with missing data.

We determined the frequency of elevated ICP by measuring the optic nerve sheath, choosing as the best cut-off value a diameter equal to or >5.8 mm. We also determined the frequency of sonographic patterns obtained by transcranial Doppler through insonation of the middle cerebral artery, to establish the cerebral ultrasound patterns we use the diagnostic correlation diagram proposed by Muñoz Sanchez et al. (21).

To evaluate the associations of clinical, mechanical ventilator, and arterial blood gas variables with ONSD ≥ 5.8 mm and PI ≥ 1.1, hypothesis tests were performed according to the nature of the independent variables. The chi-square test or Fisher's exact test was used for categorical variables, whereas Student's *t-*test or the Mann-Whitney U test was used for quantitative variables. Likewise, the associations of hemodynamic findings acquired by TCD and ONSD with mortality outcomes were evaluated.

Finally, we estimated the effect size (relative risk [RR] and 95% confidence intervals [95% CI]) by creating Poisson regression models with robust variance. Crude models were adjusted for age, sex, and PaCO_2_. Statistical significance was set at *p* < 0.05.

### Ethical considerations

The information used in this study was collected from clinical histories; therefore, informed consent was not required. The authors undertook to maintain the confidentiality of the patients, whose data were collected without identifiers, and to respect the veracity of the information. All collected information was stored in a safe place, and access was granted only to the principal investigator, who was solely responsible for safeguarding this information.

The study was conducted in accordance with the Declaration of Helsinki and Belmont Report, and patient anonymity was maintained. The research protocol was approved by the COVID-19 Research Ethics Committee of the Social Health Insurance program.

## Results

A total of 142 patients were enrolled in the study. The mean age was 51.39 ± 13.3 years, and 78.9% were men. In addition, vasopressors were used in 45.1% of the patients, and the mean MAP was 81.87 ± 10.64 mmHg. Additionally, the mean PaCO_2_ was elevated reaching 54.08 ± 16.01 mmHg.

We found that ONSD was elevated (≥5.8 mm) in 83.1% of the patients and that PI was elevated (≥1.1) in 40.1%. Moreover, the pulsatility pattern was normal in 48.6% of the patients (PI = 0.7–1.0) and was low in the rest (11.3%) (PI < 0.7). The reported mortality rate was 16.2%. Further details regarding the general characteristics of the patients are presented in Table 1.

**Table 1:** Distribution of hemodynamic parameters in patients with COVID-19 according to ONSD and PI.

			**ONSD**			**PI**		
**Variable**	**Median**	**Standard deviation**	<**5.8 (*****n** =* **24)**	≥**5.8 (*****n** =* **118)**	***P*** **-value**	<**1.1 (*****n** =* **85)**	≥**1.1 (*****n** =* **57)**	***P*** **-value**
Age^*^	51.39	13.3	47.6 (13.5)	52.2 (13.13)	0.124	51.4 (12.8)	51.3 (14.0)	0.966
**Sex**								
Female (*n*, %)	30	21.1	5 (16.7%)	25 (83.3%)	0.969	20 (66.7)	10 (33.3)	0.392
Male (*n*, %)	112	78.9	19 (17.0%)	93 (83.0%)		65 (58.1)	47 (41.9)	
Mean arterial pressure (mmHg)^*^	81.87	10.64	79.9 (10.7)	82.3 (10.6)	0.327	83.3 (10.8)	79.7 (10.1)	**0.048**
**Vasopressor use**								
No (*n*, %)	78	54.9	16 (20.5%)	62 (79.5%)	0.205	51 (65.4)	27 (34.6)	0.138
Yes (*n*, %)	64	45.1	8 (12.5%)	56 (87.5%)		34 (53.1)	30 (46.9)	
PaCO_2_ (mmHg)^±^	54.08	16.01	51.8 (10.9)	54.5 (16.9)	0.327	58.9 (17.7)	46.9 (9.2)	**<** **0.001**
SpO_2_ (%)^*^	96.89	1.88	96.1 (2.1)	97.1 (1.8)	**0.020**	96.6 (2.1)	97.4 (1.5)	**0.011**
PSV (cm/s)^*^	98.26	35.82	92.7 (27.6)	99.4 (37.3)	0.407	104.3 (33.7)	89.3 (37.4)	**0.014**
EDV (cm/s)^*^	40.04	19.46	37.3 (14.1)	40.6 (20.4)	0.449	48.4 (17.7)	27.5 (14.7)	**< 0.001**
MFV (cm/s)^*^	60.23	25.71	57.3 (19.7)	60.8 (26.8)	0.536	70.0 (24.3)	45.7 (20.4)	**< 0.001**
PI^*^	1.06	0.36	1.02 (0.3)	1.07 (0.4)	0.489	-		
ONSD (mm)^±^	6.1	0.49	-			6.1 (0.5)	6.2 (0.4)	0.331
PEEP (cmH_2_O)^*^	13.61	2.48	13.2 (2.8)	13.7 (2.4)	0.342	14.2 (2.4)	12.8 (2.3)	**< 0.001**
P_peak_ (cmH_2_O)^*^	29.47	4.62	29.3 (4.7)	29.5 (4.6)	0.835	30.5 (4.3)	27.9 (4.7)	**< 0.001**
P_plateau_ (cmH_2_O)^*^	25.7	4.31	26.3 (4.6)	25.6 (4.3)	0.461	26.5 (4.2)	24.5 (4.3)	**0.006**
P_mean_ (cmH_2_O)^*^	18.68	3.17	18.5 (3.4)	18.7 (3.1)	0.705	19.3 (2.9)	17.8 (3.3)	**0.007**
Cst (mL/cmH_2_O)^±^	36.74	12.53	33.4 (11.4)	37.4 (12.7)	0.157	34.6 (11.2)	40.0 (13.7)	**0.011**
**Outcome**								
Survived (*n*, %)	119	83.8	20 (16.8)	99 (83.2)	0.945	71 (59.7)	48 (40.4)	0.914
Died (*n*, %)	23	16.2	4 (17.4)	19 (82.6)		14 (60.8)	9 (39.1)	

^*^Expressed as mean and standard deviation. ^±^Expressed as median and range. COVID-19, coronavirus disease; PaCO_2_, partial pressure of carbon dioxide; SpO_2_, oxygen saturation; PSV, peak systolic velocity; EDV, end-diastolic velocity; MFV, mean flow velocity; PI, pulsatility index; ONSD, optic nerve sheath diameter; PEEP, positive end-expiratory pressure; P_peak_, peak airway pressure; P_plateau_, plateau pressure; P_mean_, mean airway pressure; Cst, pulmonary compliance. Values in bold are statistically significant results.

Regarding the cerebral sonographic patterns found through TCD, 24% of the patients exhibited a low velocity pattern (MFV < 40 cm/s), 22% exhibited a high velocity pattern (MFV > 79 cm/s), and the majority (54%) exhibited a normal velocity pattern (MFV = 40–79 cm/s).

The following hemodynamic patterns were found: cerebral hypoperfusion (24%), cerebral hyperperfusion (22%), normal blood flow (30%), and normalized (abnormal) blood flow with intracranial hypertension (24%).

On comparing demographic, clinical, sonographic, and ventilatory characteristics according to ONSD and PI findings, significant differences were found in oxygen saturation (SpO_2_) (difference in SpO_2_ between patients with ONSD < 5.8 mm and those ONSD ≥ 5.8 mm = 0.3%; *p* = 0.02). Similarly, on comparing gasometric (PaCO_2_ and SpO_2_), TCD (PSV, EDV, and MFV), and ventilatory (positive end-expiratory pressure [PEEP], peak airway pressure [P_peak_], plateau pressure [P_plateau_], mean airway pressure [P_mean_], and pulmonary compliance [Cst]) variables according to MAP, significant differences were found between the groups with and without elevated PI. Notably, on comparing the distribution of both ONSD and PI patterns, we found that there no significant differences between the proportions of patients who survived and those who did not (Table 1).

On evaluating associations using Poisson regression models, it was found that none of the variables were significantly associated with a greater risk of presenting with ONSD ≥ 5.8 mm. For every 10 mmHg increase in PaCO_2_, the risk of presenting with PI ≥ 1.1 decreased by 39% (95% CI: 0.50–0.74). The results related to all the mechanical ventilation variables were significant in the crude PI analysis (Table 2).

**Table 2:** Simple regression of hemodynamic parameters in patients with COVID-19 according to ONSD and PI.

	**ONSD** ≥**5.8 mm (*****n*** **=** **118)**			**PI** ≥**1.1 (*****n*** **=** **57)**		
**Variable**	**RR**	**95% CI**	***P-*** **value**	**RR**	**95% CI**	***P-*** **value**
Age (every 10 years)	1.05	0.99–1.11	0.135	0.99	0.85–1.16	0.969
Sex: Male Female(Ref.)	0.99	0.83–1.19	0.969	1.26	0.72–2.19	0.414
PaCO_2_ (every 10 mmHg)	1.02	0.99–1.05	0.275	0.61	0.50–0.74	**< 0.001**
PEEP (cmH_2_O)	1.01	0.98–1.05	0.398	0.87	0.80–0.94	**0.001**
P_peak_ (every 10 cmH_2_O)	1.02	0.87–1.20	0.833	0.47	0.31–0.73	**0.001**
P_plateau_ (every 10 cmH_2_O)	0.94	0.78–1.12	0.479	0.50	0.30–0.83	**0.007**
P_mean_ (every 5 cmH_2_O)	1.02	0.90–1.16	0.716	0.64	0.46–0.88	**0.007**
Cst (every 10 mL/cmH_2_O)	1.04	0.99–1.10	0.111	1.19	1.07–1.33	**0.001**

RR, risk ratio; CI, confidence interval; COVID-19, coronavirus disease; PaCO_2_, partial pressure of carbon dioxide; PI, pulsatility index; ONSD, optic nerve sheath diameter; PEEP, positive end-expiratory pressure; P_peak_, peak airway pressure; P_plateau_, plateau pressure; P_mean_, mean airway pressure; Cst, pulmonary compliance. Values in bold are statistically significant results.

In the multivariate analysis of hemodynamic variables according to ONSD and PI, age was associated with increased ONSD such that for every 10-year increase in age, the risk of ONSD being ≥5.8 increased by 7%. In the multiple regression analysis of PI ≥ 1.1, only PaCO_2_ maintained its statistical association as a protective factor at 36% for every 10-mmHg increase (Table 3).

**Table 3:** Multiple regression of hemodynamic parameters in patients with COVID-19 according to ONSD and PI.

	**ONSD** ≥**5.8 mm (*****n*** **=** **118)**			**IP** ≥**1.1 (*****n*** **=** **57)**		
**Variable**	**RR**	**95% CI**	***P-*** **Value**	**RR**	**95% CI**	***P-*** **value**
Age (every 10 years)	**1.07**	**1.01–1.13**	**0.042**	0.90	0.77–1.06	0.204
Sex: Male Female (Ref.)	0.91	0.76–1.10	0.312	1.39	0.79–2.44	0.252
PaCO_2_ (every 10 mmHg)	1.02	0.98–1.06	0.312	0.64	0.53–0.79	**< 0.001**
P_peak_ (every 10 cmH_2_O)	1.15	0.91–1.45	0.257	0.70	0.35–1.41	0.252
P_plateau_ (every 10 cmH_2_O)	0.73	0.50–1.08	0.114	1.61	0.71–3.64	0.252
Cst (every 10 mL/cmH_2_O)	1.04	0.96–1.12	0.382	1.07	0.92–1.24	0.395

RR, risk ratio; CI, confidence interval; COVID-19, coronavirus disease; PaCO_2_, partial pressure of carbon dioxide; PI, pulsatility index; ONSD, optic nerve sheath diameter; P_peak_, peak airway pressure; P_plateau_, plateau pressure; P_mean_, mean airway pressure; Cst, pulmonary compliance. Values in bold are statistically significant results.

On comparing demographic, clinical, sonographic, and ventilatory characteristics according to mortality, significant differences were found in the use of vasopressors and hemoglobin (Hb) levels (difference in Hb between surviving and deceased patients = 1.7; *p* < 0.001). Similarly, significant differences were found in the TCD variables (PSV and MFV), as well as in the ventilator variables (PEEP, P_peak_, P_mean_, and Cst except for in P _Plateau_; Table 4).

**Table 4:** Hemodynamic patterns and mortality in patients with COVID-19.

	**Mortality**		
	**No (*****n** =* **119)**	**Yes (*****n** =* **23)**	***P-*** **Value**
Age	50.8 (12.9)	24 (14.7)	0.251
**Sex**			
Female	24 (80.0)	6 (20.0)	0.524
Male	95 (84.8)	17 (15.2)	
Mean blood pressure (mmHg)^*^	81.8 (10.5)	82.3 (11.8)	0.847
**Vasopressor use**			
No	71 (91.0)	7 (9.0)	**0.01**
Yes	48 (75.0)	16 (25.0)	
etCO${\;}_{2}^{\pm }$	45 (39–54)	45.5 (40–51)	0.9477
PaCO_2_ (mmHg)^±^	50.5 (43.8–60)	53.2 (41.8–58.3)	0.801
pH^*^	7.37 (0.8)	7.34 (0.1)	0.216
PaO_2_ (mmHg)^±^	98 (84.1–116)	98 (83.9–120)	0.971
SatO_2_ (%)^*^	96.8 (1.7)	96.6 (1.9)	0.733
SpO_2_ (%)^*^	96.9 (1.9)	96.8 (1.9)	0.766
Temperature (°C)^*^	36.3 (0.8)	36.7 (0.9)	0.12
Hb (g/dL)^*^	11.9 (1.9)	10.2 (1.6)	**< 0.001**
PSV (cm/s)^*^	95.5 (35.6)	112.7 (35.9)	**0.035**
EDV (cm/s)^*^	39.2 (19.3)	44.6 (20.3)	0.225
MFV (cm/s)^*^	58.2 (24.6)	70.9 (28.9)	**0.029**
PI^*^	1.06 (0.3)	1.05 (0.4)	0.769
ONSD (mm)^±^	6.1 (0.5)	6.0 (0.4)	0.571
PEEP (cmH_2_O)^*^	13.4 (2.3)	14.8 (2.9)	**0.009**
P_peak_ (cmH_2_O)^*^	29.1 (4.6)	31.8 (4.1)	**0.007**
P_plateau_ (cmH_2_O)^*^	25.2 (4.1)	28.3 (4.3)	0.717
P_mean_ (cmH_2_O)^*^	18.2 (2.8)	21.1 (3.6)	**0.001**
Cst (mL/cmH_2_O)^±^	35.7 (29–44.2)	29.6 (28.1–40.2)	0.052

^*^P-values obtained by Student's *t*-test. ^±^*P*-values obtained by rank test. COVID-19, coronavirus disease; etCO_2_, end-tidal carbon dioxide; PaCO_2_, partial pressure of carbon dioxide; PaO_2_, arterial oxygen partial pressure; SatO_2_, peripheral oxygen saturation; SpO_2_, oxygen saturation; Hb, hemoglobin; PSV, peak systolic velocity; EDV, end-diastolic velocity; MFV, mean flow velocity; PI, pulsatility index; ONSD, optic nerve sheath diameter; PEEP, positive end-expiratory pressure; P_peak_, peak airway pressure; P_plateau_, plateau pressure; P_mean_, mean airway pressure; Cst, pulmonary compliance. Values in bold are statistically significant results.

On evaluating associations using Poisson regression models, SPV and MFV were identified as risk factors for mortality in the crude analysis. In the multivariate analysis of hemodynamic variables according to mortality, MFV had an adjusted RR of 1.15, implying that for every 1-unit increase in MFV, the risk of mortality increased by 15% (95% CI: 1.03–1.28; Table 5).

**Table 5:** Simple and multiple regression of the associations of hemodynamic parameters and ONSD with mortality in patients with COVID-19.

	**Simple regression**			**Multiple regression**		
	**RRc**	**95% CI**	***P-*** **value**	**RRa**	**95% CI**	***P-*** **value**
**Age**	1.02	0.99–1.05	0.286	1.02	0.99–1.05	0.235
**Sex**						
**Female**	Ref.			Ref.		
**Male**	0.76	0.33–1.76	0.521	0.74	0.33–1.65	0.462
**PSV (every 10 cm/s)**	1.10	1.02–1.20	**0.022**			
**EDV (every 10 cm/s)**	1.12	0.95–1.33	0.193			
**MFV (cm/s)**	1.15	1.03–1.29	**0.011**	**1.15**	**1.03–1.28**	**0.010**
**PI**	0.85	0.28–2.61	0.778			
**ONSD (mm)**	0.80	0.41–1.55	0.508			

COVID-19, coronavirus disease; RRc, crude relative risk; RRa, adjusted relative risk; PSV, peak systolic velocity; EDV, end-diastolic velocity; MFV, mean flow velocity; PI, pulsatility index; ONSD, optic nerve sheath diameter. Values in bold are statistically significant results.

## Discussion

The present study was conducted with the objective of describing neurosonological and ultrasound findings related to cerebral hemodynamics acquired using TCD and ONSD measurement in patients with severe COVID-19 hospitalized in the intensive care unit of a national referral hospital in Peru; additionally, factors associated with neurosonological and ultrasound findings related to intracranial hypertension and the association of cerebral hemodynamic variables and ONSD with mortality were also explored.

The most relevant finding of this study was that a very large proportion (83%) of patients who were admitted with acute respiratory failure due to severe COVID-19 and were on mechanical ventilation presented with ONSD ≥ 5.8 mm. In addition, it is important to highlight the following findings: of the 142 patients included in this study, 23 patients (16%) died, and of these 23 deceased patients, four died due to intracranial hypertension resulting in brain death.

ONSD has been widely studied as a useful, reliable, and non-invasive measure for estimating ICP. The accuracy of ONSD for the detection of elevated ICP was evaluated through a meta-analysis, where the pooled area under the receiver operating characteristic (ROC) curve for ONSD was reported to be 0.94 (22), 0.93, (17) and 0.94 (23) by different studies. A significant linear correlation was found between ONSD and ICP; therefore, the diagnostic accuracy of ONSD acquired via ultrasound was found to be good when used to detect intracranial hypertension (24, 25).

However, there is no consensus regarding the optimal ONSD threshold for detecting elevated ICP. Wang et al. found that extended ONSD was a strong predictor of elevated ICP; an ONSD cutoff of 5.83 mm indicated an estimated ICP of > 22 mmHg with a sensitivity of 94.4% and specificity of 81.0% (26). Geeraerts reported that 5.86 mm is the ideal ONSD cutoff value (sensitivity, 95%; specificity, 79%) for detecting elevated ICP, (25) and Robba found that the ideal cutoff value for predicting ICP > 20 mmHg was 5.85 mm (27). Therefore, we chose the cutoff value of 5.8 mm in order to estimate the frequency of intracranial hypertension in our study.

An important finding of this study was the lack of an association between elevated carbon dioxide (CO_2_) levels and ONSD. Patients who underwent ocular ultrasound had acute respiratory distress syndrome caused by SARS-CoV-2 and were on mechanical ventilation with low tidal volumes (protective mechanical ventilation) (28–31), which resulted in increased arterial CO_2_ pressure (PaCO_2_).

The mean PaCO_2_ in the study population was 54 mmHg. We first considered that this permissive hypercapnia could be a physiological cause of the increase in ONSD in these patients, as the arteriolar vasodilator effect would lead to an increase in CBF, which in turn would increase ICP. However, on evaluating the RR between PaCO_2_ and ONSD, we found no risk, with a non-significant RR of 1.02 (95% CI: 0.98–1.06; *p* = 0.312); therefore, we established that the increase in ONSD was not associated with the hypercapnia due to which these patients underwent mechanical ventilation.

On the other hand, the patients were subjected to high levels of PEEP, with an average PEEP level of 14 ± 3 mmHg. These elevated PEEP levels lead to an increase in mean airway pressure [P_mean_], and intrathoracic positive pressure levels, resulting in a decrease in venous return to the heart, with a consequent decrease in cerebral venous drainage and an increase in ICP.

However, the risk analysis did not reveal any relationship between positive intrathoracic pressures (PEEP, P_m_, P_plateau_, and P_peak_) and ONSD ≥ 5.8 mm; therefore, the finding of increased ONSD was not associated with the levels of PEEP, Pm, P_plateau_, P_peak_, C_st_, and PaCO_2_.

Based on the above findings, we can conclude that 83% of our patients with severe COVID-19 on mechanical ventilation exhibited an increase in ICP, as indicated by the ONSD measurement, and that this increase was due to COVID-19 and was not associated with an external factor resulting from the treatment administered. Whether this increase in ICP in patients with severe COVID-19 was due to direct invasion of the virus into the central nervous system or whether it was a secondary development to a systemic inflammatory response could not be determined in this study, and further studies are needed to clarify this.

With regard to the TCD findings, we used a correlation diagram between the ultrasound patterns and cerebral hemodynamic status in our patients, as proposed by Muñoz Sánchez et al. (21) The most striking finding was that 24% of the patients exhibited low velocity echographic patterns, which translates into hemodynamic patterns indicative of low CBF or cerebral hypoperfusion; notably, decreased CBF was found in the absence of arterial hypotension or shock, and a systemic cause for this decrease was not found.

To add to the finding that 83% of patients had increased ICP, as estimated through ONSD, we could suggest that the cause of the decrease in CBF was elevated ICP. Helms et al. found that 100% of patients with severe COVID-19 on mechanical ventilation had cerebral hypoperfusion at the temporal and frontal levels (4).

Another important TCD finding was that 48% of our patients had a high pulsatility pattern; however, this percentage was underestimated because PI values are intimately influenced by PaCO_2_ levels, as the higher the PaCO_2_, the lower the PI (32). Therefore, if these patients were not managed with permissive hypercapnia and were instead in normocapnia, the proportion of patients with elevated PI would have been larger.

There are various causes of elevated PI, including intracranial hypertension (33), hypocapnia (21, 32), microangiopathy (34, 35), intracranial vascular stenosis (36), and brain death (37). Thus, an increase in PI does not represent an increase in ICP in every patient. Hence, TCD is not a reliable method to estimate increased ICP, and its usefulness is inferior to that of ONSD (22, 38). However, the frequency of patients with severe COVID-19 on mechanical ventilation having elevated PI was high, which supports the ONSD-based findings.

Twenty-two percent of our patients exhibited an increased mean velocity (VFM ≥ 80 cm/s). Furthermore, we found that increased MFV and systolic velocity were associated with an increased risk of mortality in the simple regression analysis; one explanation for this is that the hyperemia could be reactive to acute endothelial dysfunction, cerebral vascular vasodilatation, and/or sepsis-induced impairment of cerebral autoregulation (39). Therefore, the magnitude of increased MFV would reflect a greater level of systemic inflammation secondary to SARS-CoV-2 sepsis.

In the multiple regression analysis, MFV was found to be a risk factor for mortality (RR: 1.15), which can be explained by a potential relationship between the systemic inflammatory response caused by COVID-19 and increased velocities in cerebral arteries; this deterioration of CBF may be due to the release of inflammatory mediators, as evidenced in other critical conditions.

However, regarding the relationship between PaCO_2_ levels and cerebral artery velocities, our study demonstrated a close inversely proportional relationship between PaCO_2_ levels and PI (*p* < 0.001): the higher the PaCO_2_ level, the lower the PI. This causes a fall in cerebral vascular resistance and, therefore, an increase in MFV of the cerebral arteries.

Previously published studies have addressed the issue of neurosonological findings in critically ill patients with COVID-19 and their clinical outcomes (40–42); our study, in addition to describing the neurosonological findings, studied the association of hemodynamic and ventilatory parameters with neurosonological findings and mortality. Recording ventilatory mechanics to interpret the results is essential because intrathoracic pressures are very high and the lungs are very damaged, this could cause a decrease in cerebral venous return and increase ICP, therefore, reliable conclusions cannot be obtained without analyzing the relationship between these variables.

The main limitation of this study was that the estimation of ICP was performed non-invasively, and the methods used were operator-dependent. In addition, most enrolled patients had hypercapnia, which could limit the generalizability of the conclusions of our study. Subsequent studies on a population of mechanically ventilated patients with normocapnia, which involve invasive ICP measurements, are needed to corroborate our findings. The present study has some strengths. The study population comprised a well-selected group of critical patients on mechanical ventilation in the acute phase of COVID-19, and ventilatory and arterial gas parameters were recorded and simultaneously analyzed at the time of the neurosonology examinations; this increases internal validity and reduces risk of bias.

## Conclusions

A very high rate of intracranial hypertension was found in patients with severe COVID-19, as estimated using noninvasive ultrasound and neurosonology techniques, with ONSD measurement being the most reliable method. This study allows us to establish that the increase in ICP measured by ONSD in patients with severe COVID-19 on mechanical ventilation is not associated to hypercapnia or elevated intrathoracic pressures derived from protective mechanical ventilation.

## Data availability statement

The raw data supporting the conclusions of this article will be made available by the authors, without undue reservation.

## Author contributions

OH-O: Conceptualization, Data curation, Investigation, Methodology, Project administration, Resources, Supervision, Writing–original draft, Writing–review & editing. MV: Data curation, Methodology, Software, Supervision, Writing–original draft, Writing–review & editing. VF-R: Data curation, Formal analysis, Methodology, Software, Writing–original draft, Writing–review & editing. DF-M: Data curation, Investigation, Methodology, Software, Writing–original draft, Writing–review & editing. RL-C: Project administration, Resources, Supervision, Validation, Writing–original draft, Writing–review & editing. RV: Data curation, Investigation, Project administration, Resources, Supervision, Writing–original draft, Writing–review & editing.
